# Association of β-Amyloid Burden With Sleep Dysfunction and Cognitive Impairment in Elderly Individuals With Cognitive Disorders

**DOI:** 10.1001/jamanetworkopen.2019.13383

**Published:** 2019-10-16

**Authors:** Jason C. You, Erica Jones, Devon E. Cross, Abigail C. Lyon, Hyunseung Kang, Andrew B. Newberg, Carol F. Lippa

**Affiliations:** 1 Cognitive Disorders and Comprehensive Alzheimer’s Disease Center, Department of Neurology, Thomas Jefferson University Hospital, Philadelphia, Pennsylvania; 2 Sidney Kimmel Medical College, Thomas Jefferson University, Philadelphia, Pennsylvania; 3 Department of Internal Medicine, Lankenau Medical Center, Wynnewood, Pennsylvania; 4 Department of Statistics, University of Wisconsin-Madison, Madison; 5 Marcus Institute for Integrative Health, Department of Integrative Medicine, Thomas Jefferson University Hospital, Philadelphia, Pennsylvania; 6 Department of Radiology, Thomas Jefferson University Hospital, Philadelphia, Pennsylvania

## Abstract

**Importance:**

Evidence shows that sleep dysfunction and β-amyloid (Aβ) deposition work synergistically to impair brain function in individuals with normal cognition, increasing the risk of developing dementia later in life. However, whether Aβ continues to play an integral role in sleep dysfunction after the onset of cognitive decline in individuals with dementia is unclear.

**Objective:**

To determine whether Aβ deposition in the brain is associated with subjective measures of sleep quality and cognition in elderly individuals with cognitive disorders.

**Design, Setting, and Participants:**

A nested survey study was conducted at the Cognitive Disorders and Comprehensive Alzheimer Disease Center of Thomas Jefferson University Hospital in Philadelphia, Pennsylvania. Participants included patients aged 65 years and older with cognitive disorders verified by neuropsychological testing. Eligible participants were identified from a referral center–based sample of patients who underwent fluorine 18–labeled florbetaben positron emission tomography imaging at Thomas Jefferson University Hospital as part of the multicenter Imaging Dementia-Evidence for Amyloid Scanning study. Data collection and analysis occurred between November 2018 and March 2019.

**Main Outcomes and Measures:**

Sleep quality was measured via responses to sleep questionnaires, Aβ deposition was measured via fluorine 18–labeled florbetaben positron emission tomography, and cognition was measured via Mini-Mental State Examination (MMSE) performance.

**Results:**

Of the 67 eligible participants, 52 (77.6%) gave informed consent to participate in the study. Of the 52 enrolled participants (mean [SD] age, 76.6 [7.4] years), 27 (51.9%) were women. Daytime sleepiness was associated with Aβ deposition in the brainstem (*B* = 0.0063; 95% CI, 0.001 to 0.012; *P* = .02), but not MMSE performance (*B* = −0.01; 95% CI, −0.39 to 0.37; *P* = .96). The number of nocturnal awakenings was associated with Aβ deposition in the precuneus (*B* = 0.11; 95% CI, 0.06 to 0.17; *P* < .001) and poor MMSE performance (*B* = −2.13; 95% CI, −3.13 to −1.13; *P* < .001). Mediation analysis demonstrated an indirect association between Aβ deposition and poor MMSE performance that relied on nocturnal awakenings as an intermediary (*B* = −3.99; 95% CI, −7.88 to −0.83; *P* = .01).

**Conclusions and Relevance:**

Nighttime sleep disruption may mediate the association between Aβ and cognitive impairment, suggesting that there is an underlying sleep-dependent mechanism that links Aβ burden in the brain to cognitive decline. Further elucidation of this mechanism may improve understanding of disease processes associated with Aβ accumulation.

## Key Points

**Question:**

Is β-amyloid deposition in the brain associated with sleep dysfunction and cognition in elderly individuals with cognitive disorders?

**Findings:**

In this survey study of 52 participants aged 65 years and older, β-amyloid deposition in the precuneus was associated with the number of nocturnal awakenings, whereas β-amyloid deposition in the brainstem was associated with daytime sleepiness. Nocturnal awakenings, but not daytime sleepiness, were associated with poor cognition, and β-amyloid deposition was indirectly associated with cognitive impairment via nocturnal awakenings.

**Meaning:**

In elderly individuals with cognitive disorders, a mechanism that involves disruption of nighttime sleep may underlie the association between β-amyloid deposition and cognitive impairment.

## Teaser

This survey study examines whether β-amyloid deposition in the brain is associated with sleep dysfunction and cognition in elderly individuals with cognitive disorders.

## Introduction

Sleep dysfunction is associated with cognitive decline in the aging population.^1,2^ Increasing evidence shows that sleep and circadian rhythm disturbances predispose the brain to accumulation of β-amyloid (Aβ),^1,3^ a protein metabolite that impairs neuronal function and is linked to numerous cognitive disorders, including Alzheimer disease (AD), dementia with Lewy bodies, Parkinson disease dementia, cerebrovascular dementia, and frontotemporal dementia.^4,5^ Studies^6,7,8,9,10,11,12^ in humans have shown that a variety of poor sleep indicators, including prolonged sleep latency, increased sleep fragmentation, decreased total sleep time, and excessive daytime sleepiness, are associated with increased Aβ deposition in the brain. Mechanistic studies have also demonstrated that sleep disruption promotes Aβ accumulation by simultaneously upregulating Aβ synthesis^13,14^ and interfering with Aβ clearance.^14,15^

Importantly, the relationship between sleep dysfunction and Aβ appears to be bidirectional because elevated levels of Aβ in the brain also impair slow-wave sleep,^16^ thereby exacerbating sleep problems.^17,18^ The synergistic relationship between Aβ abnormalities and sleep dysfunction has been shown to interfere with memory consolidation and recall.^9,16^ Moreover, individuals with normal cognition experiencing poor sleep quality have increased brain Aβ burden and are at higher risk of developing dementia later in life.^8,10,19,20^

Notably, patients with cognitive disorders have profound disturbances in sleep architecture and circadian rhythms.^2,21^ However, studies examining the association between Aβ and sleep have, thus far, largely been performed in individuals with normal cognition, and whether Aβ continues to play an important role in sleep dysfunction after the onset of cognitive decline is unclear. Therefore, this study aims to examine the associations between Aβ abnormalities, subjective measures of sleep quality, and cognitive function in elderly individuals with cognitive disorders.

## Methods

### Participants

All participants in this study were patients with cognitive disorders who received care at the Cognitive Disorders and Comprehensive Alzheimer Disease Center at Thomas Jefferson University Hospital, a major referral center in the Delaware Valley. The center is an outpatient clinic that serves patients who are living independently, with family or caregivers, or in assisted living facilities. Therefore, hospitalized patients or those living in nursing homes were not included in the study, even though there were no specific exclusion criteria for these conditions. Inclusion criteria included age 65 years or older and a diagnosis of mild cognitive impairment (MCI) or dementia based on criteria established by the *Diagnostic and Statistical Manual of Mental Disorders* (Fifth Edition) and/or the National Institutes of Aging and the Alzheimer Association. Patients with normal cognition or subjective complaints unverified by cognitive testing were excluded from the study. Other major exclusion criteria included cancer requiring active therapy, hip or pelvic fracture within the 12 months before enrollment, and loss to follow-up. A sample of 80 patients who underwent Aβ positron emission tomography (PET) imaging at Thomas Jefferson University Hospital as part of the multicenter Imaging Dementia-Evidence for Amyloid Scanning Study (ClinicalTrials.gov identifier: NCT02420756) were originally considered for this study. Of this sample, 67 patients were deemed eligible for the study on the basis of the aforementioned criteria.

This study was approved by the institutional review board at Thomas Jefferson University Hospital and was categorized as minimal risk. Verbal informed consent was obtained from participants as they were contacted via phone for the study. The informed consent process included education about protected health information, the purpose of the study, data to be gathered by the investigators, risks of the study, steps taken to ensure participant privacy and confidentiality, and information on how to withdraw consent. Participants were told that there was no monetary compensation for the study. This study follows the Strengthening the Reporting of Observational Studies in Epidemiology (STROBE) reporting guideline.^22^

Data collection and analysis occurred between November 2018 and March 2019. Data pertaining to race/ethnicity were extracted from electronic health records and were based on the participants’ own responses to previous health questionnaires. Race/ethnicity was subsequently assessed as a potential confounding factor in the analyses.

### Cognitive Assessment

Cognition was assessed via the participants’ most recent performance on the Mini-Mental State Examination (MMSE). The MMSE is a 30-point questionnaire that is widely used to evaluate the severity of cognitive impairment in individuals with cognitive disorders.^23^ The questionnaire tests multiple cognitive domains, including orientation, attention and calculation, language, verbal memory, and visuospatial planning. Score cutoffs for the MMSE are as follows: greater than or equal to 24 for normal cognition, between 19 and 23 for MCI, between 10 and 18 for moderate cognitive impairment, and less than or equal to 9 for severe cognitive impairment.^24^

### Sleep Quality Assessment

Eligible participants and their family or caregivers were contacted via phone for sleep quality assessment; 52 participants (77.6%) gave verbal informed consent to participate in the study. Reasons for refusing consent included not wanting to share personal information over the phone (6 participants [40.0%]), inconvenient timing (3 participants [20.0%]), inaccessible after 3 or more attempts (3 participants [20.0%]), no longer following up with a clinician at Thomas Jefferson University Hospital (2 participants [13.3%]), and patient was deceased (1 participant [6.7%]). Sleep quality was assessed via 2 sleep questionnaires that have previously been validated (eAppendix in the Supplement).^19,25^ We made certain that participants with MMSE scores less than 24 had at least 1 family member or caregiver present during the questionnaire to corroborate their responses; those with higher scores were also encouraged to respond jointly with family members to improve response accuracy.

### Brain Imaging and Regional Analysis

For Aβ PET imaging, participants were injected intravenously with fluorine 18–labeled florbetaben tracer (Neuraceq; Piramel Imaging), and imaging was performed approximately 60 minutes later as per a standard protocol using either PET–magnetic resonance imaging or PET–computed tomography. Image processing was performed using MIMNeuro software (version 6.6, MIM Software), and Aβ deposition in different brain regions was calculated via standardized uptake value ratio. Regions of interest (ROIs) were based on susceptibility to Aβ deposition^19,26^ and included the anterior and posterior cingulate gyrus, medial frontal lobe, medial and lateral temporal lobe, precuneus, superior parietal lobe, thalamus, and brainstem.

### Statistical Analysis

The deposition of Aβ across ROIs was analyzed via the Welch analysis of variance with the Games-Howell post hoc test, which helps to detect differences between samples with unequal variances. Associations between Aβ deposition, different sleep measures, and MMSE performance were analyzed via multiple linear regression. Data in regression analyses were modeled by least-squares regression lines, with each line having a slope equivalent to the regression coefficient (*B*), also known as regression weight. A Wald-type 95% CI for *B* was then calculated from the SE of the sampling distribution of data points from the slope of the regression line. The strength of association between variables of interest was determined by calculating the coefficient of determination (*R*^2^). Forward stepwise regression was used to control for potentially confounding variables; this multivariate regression method incorporates variables in stepwise fashion until the model fit is no longer significantly improved via the F-test. Therefore, variables that make statistically significant contributions to the multivariate regression are distinguished from those that do not. The problem of multiple comparisons was encountered when correlating data involving Aβ and was addressed via 2 methods: Bonferroni corrections for α levels and performing a limited number of planned comparisons for specific ROIs based on hypotheses. It is important to mention that Aβ deposition exhibits a high degree of collinearity across different cortical regions^27^; therefore, corrections for multiple comparisons should be interpreted with caution because they can increase the risk of a type II error.^28^ Comparative and regression analyses were performed using SPSS statistical software version 25 (IBM). Unless otherwise stated, 2-sided hypothesis testing was conducted, with type I error controlled at α = .05.

Mediation analysis was used to evaluate whether the association between Aβ deposition and poor MMSE performance could be mediated by nocturnal awakenings. Mediation analysis is a multivariate regression assay that uses a series of regressions to assess the strength of association between 2 variables of interest (*X* and *Y*) under different conditions.^29,30^ These conditions are defined by the presence or absence of a third variable (*M*) that is hypothesized to mediate the association between *X* and *Y*. Regressions performed for mediation analysis can be represented graphically by a pathway diagram that illustrates the relationship between *X* and *Y* with and without considering *M* (ie, the indirect and direct pathways, respectively). By assessing the degree of influence that *M* has on the association between *X* and *Y*, a regression coefficient for the mediation effect of *M* can thus be calculated. In summary, mediation analysis assesses whether the association between 2 variables is better explained by direct association (ie, does not require mediation) or indirect association (ie, requires mediation by another variable). Matrix calculations and bootstrapping for mediation analysis was performed via the PROCESS (version 3.3) macro for SPSS.^29^

## Results

Overall, 52 participants (mean [SD] age, 76.6 [7.4] years) were included in the analyses (Table). Among the 27 female participants (51.9%), the mean (SD) age was 77.8 (6.4) years (age range, 66-91 years). Among the 25 male participants (48.1%), the mean (SD) age was 75.4 (8.3) years (age range, 66-94 years). Among the female participants, 24 were white (88.9%), 2 were African American (7.4%), and 1 was Hispanic (3.7%). Among the male participants, 22 were white (88.0%), 2 were African American (8.0%), and 1 was Hispanic (4.0%). Using a standardized uptake value ratio threshold of 1.2,^31^ elevated brain Aβ deposition was detected in 34 of the 52 participants (65.4%), with levels of Aβ being highest in the precuneus, anterior cingulate gyrus, and brainstem relative to other brain regions (eFigure in the Supplement). Of the participants with elevated brain Aβ deposition, 16 (47.1%) were female and 18 (52.9%) were male. We found no significant difference in tracer detection between participants who underwent PET–magnetic resonance imaging vs PET–computed tomography (*t*_5_ = −0.22; *P* = .83).

**Table.** Participant Demographic and Clinical Characteristics

Chracteristic^a^	Participants, No. (%) (N = 52)
Demographic characteristics	
Age, mean (SD), y	76.6 (7.4)
Female	27 (51.9)
Race/ethnicity	
White	46 (88.5)
African American	4 (7.7)
Hispanic or Latino	2 (3.8)
Diagnosis	
Alzheimer disease	21 (40.4)
Cerebrovascular dementia	4 (7.7)
Dementia with Lewy bodies	5 (9.6)
Parkinson disease dementia	1 (1.9)
Frontotemporal dementia	4 (7.7)
Amnestic mild cognitive impairment	7 (13.5)
Nonamnestic mild cognitive impairment	3 (5.8)
Mixed dementia	7 (13.5)
Alzheimer disease and cerebrovascular dementia	6 (11.5)
Hippocampal sclerosis and cerebrovascular dementia	1 (1.9)
Mini-Mental State Examination score, median (IQR)	24 (18.3-26.8)
Time elapsed since Mini-Mental State Examination, median (IQR), mo	13 (6-20)
β-Amyloid deposition, mean (SD), standardized uptake value ratio	1.2 (0.16)
Use of dementia medication^b^	34 (65.4)
Sleep characteristics	
Latency to sleep, median (IQR), min^c^	8.8 (5-18.1)
Total nighttime sleep, mean (SD), h	8.4 (2.3)
Nocturnal awakenings, median (IQR), No.	2 (1-2.5)
Difficulty returning to sleep ≥1 d/wk^c^	16 (34)
Snore	27 (51.9)
Sleep apnea	11 (21.1)
Use of sleep medication^d^	13 (25)
Overall nighttime sleep quality score, median (IQR)^e^	8 (6-8)
Daytime sleepiness score, mean (SD)^e^	7.8 (5)

Abbreviation: IQR, interquartile range.

^a^ Data with normal distributions are reported using mean (SD); data with nonnormal distributions are reported using median (IQR).

^b^ Dementia medications include donepezil, rivastigmine, and memantine.

^c^ The quality of the data was poor, with no response from more than 10% of participants.

^d^ Sleep medications include benzodiazepines, Z-drugs, mirtazapine, trazodone, quetiapine, tricylic antidepressants, first-generation anticholinergics, and melatonin.

^e^ Sleep quality and daytime sleepiness scores were assessed via 2 sleep questionnaires that have previously been validated (eAppendix in the Supplement).^19,25^ Participants rated their sleep quality on a scale of 0 (terrible) to 10 (excellent). A daytime sleepiness score of 10 or greater indicates excessive daytime sleepiness.

### Daytime Sleepiness, Aβ Deposition, and MMSE Performance

**Figure 1.**
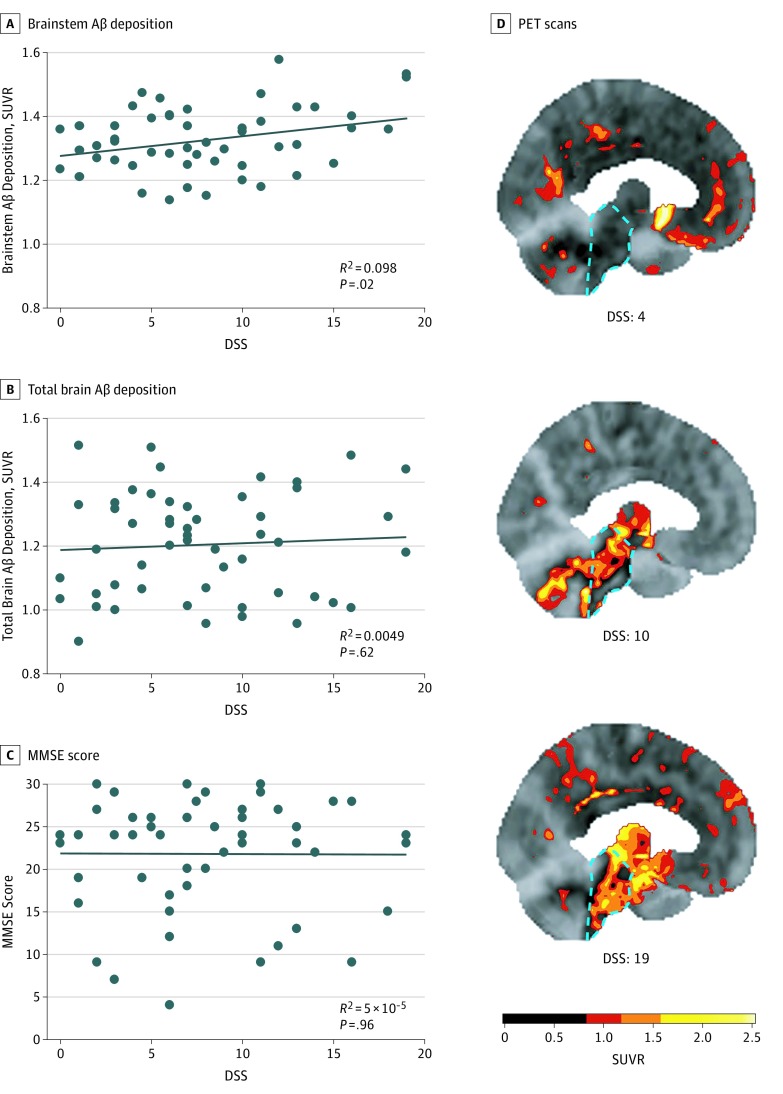
Associations of Daytime Sleepiness Score (DSS) With β-Amyloid (Aβ) Deposition and Mini-Mental State Examination (MMSE) Performance A-C, The DSS was associated with brainstem Aβ deposition (*B* = 0.0063; 95% CI, 0.001 to 0.012; *P* = .02) (A), but not with total brain Aβ deposition (*B* = 0.0023; 95% CI, −0.007 to 0.011; *P* = .62) (B) or MMSE scores (*B* = −0.01; 95% CI, −0.39 to 0.37; *P* = .96) (C). Data points represent individual patients. D, Representative fluorine 18–labeled florbetaben Aβ positron emission tomography (PET) scan images from 3 different patients with DSSs of 4, 10, and 19, respectively, are shown. A DSS of 10 or greater indicates excessive daytime sleepiness. The location of the brainstem in each scan is highlighted by a dashed cyan outline. Scale bar indicates units of standardized uptake value ratio (SUVR), with higher SUVRs indicating higher levels of Aβ deposition.

In this sample of individuals with cognitive disorders, we observed a significant positive association between daytime sleepiness scores and Aβ deposition in the brainstem (*B* = 0.0063; 95% CI, 0.001 to 0.012; *P* = .02) (Figure 1A and D). However, daytime sleepiness was not associated with Aβ burden in any other ROI, as demonstrated by the nonsignificant association between daytime sleepiness and total brain Aβ deposition (*B* = 0.0023; 95% CI, −0.007 to 0.011; *P* = .62) (Figure 1B). Moreover, there was no significant association between daytime sleepiness and MMSE performance (*B* = −0.01; 95% CI, −0.39 to 0.37; *P* = .96) (Figure 1C).

### Nocturnal Awakenings, Aβ Deposition, and MMSE Performance

**Figure 2.**
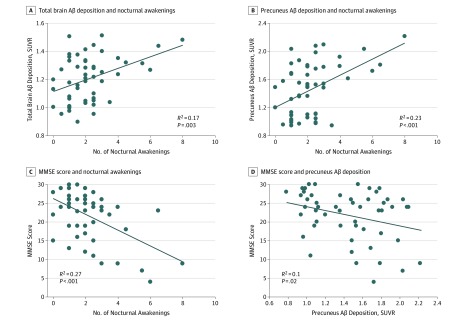
Associations of Nocturnal Awakenings With β-Amyloid (Aβ) Deposition and Mini-Mental State Examination (MMSE) Performance Association of reported number of nocturnal awakenings in a typical night with total brain Aβ deposition (*B* = 0.041; 95% CI, 0.015 to 0.067; *P* = .003) (A), association of nocturnal awakenings with Aβ deposition in the precuneus (*B* = 0.11; 95% CI, 0.06 to 0.17; *P* < .001) (B), association of nocturnal awakenings with MMSE scores (*B* = −2.13; 95% CI, −3.13 to −1.13; *P* < .001) (C), and association of Aβ deposition in the precuneus with MMSE scores (*B* = −5.54; 95% CI, −10.24 to −0.85; *P* = .02) (D) are shown. Data points represent individual patients. Trendlines indicate significant regressions. SUVR indicates standardized uptake value ratio.

The number of nocturnal awakenings reported by participants demonstrated a significant positive association with total brain Aβ deposition (*B* = 0.041; 95% CI, 0.015 to 0.067; *P* = .003) (Figure 2A). Subsequent regional analysis revealed that nocturnal awakenings had the strongest association with Aβ deposition in the precuneus (*B* = 0.11; 95% CI, 0.06 to 0.17; *P* < .001) (Figure 2B) vs other brain regions (eTable in the Supplement). Nocturnal awakenings also demonstrated a significant negative association with MMSE performance (*B* = −2.13; 95% CI, −3.13 to −1.13; *P* < .001) (Figure 2C). Notably, even after controlling for participant age, sex, race/ethnicity, total hours of nighttime sleep, sleep apnea, sleep medication use, and dementia medication use, both the association between precuneus Aβ deposition and nocturnal awakenings (*B* = 0.095; 95% CI, 0.023 to 0.17; *P* = .01) and the association between nocturnal awakenings and poor MMSE performance (*B* = −1.64; 95% CI, −2.8 to −0.48; *P* = .01) remained significant. We next assessed whether Aβ deposition had a direct association with MMSE performance and found a significant but weak inverse association (*B* = −5.54; 95% CI, −10.24 to −0.85; *P* = .02) (Figure 2D). The inverse association between Aβ and MMSE performance was no longer significant after controlling for participant age (*B* = −3.07; 95% CI, −7.6 to 1.46; *P* = .18).

Because Aβ is thought to primarily drive disease progression in AD, we also tested associations between precuneus Aβ deposition, nocturnal awakenings, and MMSE performance in a subset of 34 participants with AD or amnestic MCI, a prodromal stage of AD.^32^ Similar to our overall findings, nocturnal awakenings were positively associated with precuneus Aβ deposition (*B* = 0.097; 95% CI, 0.042 to 0.15; *P* = .001) and negatively associated with MMSE performance (*B* = −1.86; 95% CI, −3.00 to −0.72; *P* = .002) in patients with AD or amnestic MCI. However, Aβ deposition and poor MMSE performance were not significantly associated in this subgroup (*B* = −6.26; 95% CI, −13.19 to 0.62; *P* = .07).

### Mediation Analysis of Aβ Deposition, Nocturnal Awakenings, and MMSE Performance

Given the much stronger association of Aβ deposition with nocturnal awakenings vs poor MMSE performance, we hypothesized that Aβ did not have a direct association with poor cognition but instead was associated with cognitive impairment indirectly via nocturnal awakenings as an intermediary. This hypothesis is supported by previous studies^9,16^ showing that sleep modulates the association between Aβ and cognition in individuals with normal cognition. To test whether a modulatory association of sleep with Aβ and cognition could also be present in individuals with cognitive disorders, we used mediation analysis to assess whether nocturnal awakenings mediated the association between Aβ and poor MMSE performance in our sample of participants. A full description of mediation analysis is provided in the Statistical Analysis subsection of the Methods section.

**Figure 3.**
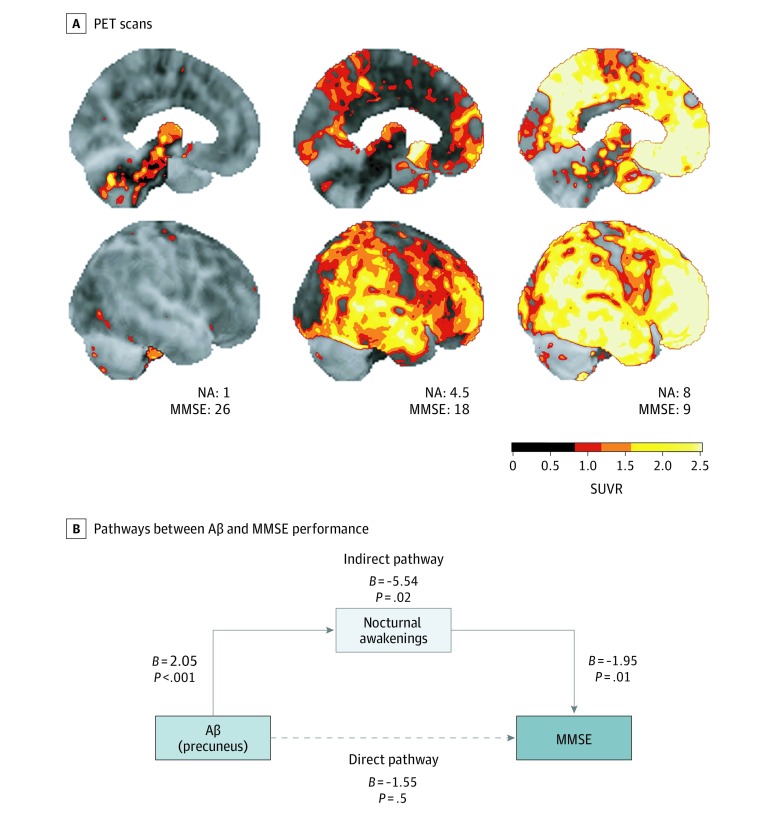
Mediation Analysis of Associations Between β-Amyloid (Aβ) Deposition, Nocturnal Awakenings (NAs), and Mini-Mental State Examination (MMSE) Performance A, Representative Aβ positron emission tomography (PET) scan images from 3 different patients who report a mean of 1, 4.5 (ie, 4-5), and 8 NAs are shown. The MMSE scores are also displayed. Top and bottom rows of images show left midsagittal and right lateral views of the brain, respectively. Scale bar indicates units of standardized uptake ratio value (SUVR), with higher SUVRs indicating higher levels of Aβ deposition. B, The direct pathway between Aβ deposition in the precuneus and MMSE performance is not significant (*B* = −1.55; 95% CI, −6.42 to 3.32; *P* = .50). However, an indirect pathway connecting Aβ deposition in the precuneus to poor MMSE performance via NAs is significant (*B* = −5.54; 95% CI, −10.24 to −0.85; *P* = .02). Nocturnal awakening is a significant mediator of the association between Aβ and poor MMSE performance (*B* = −3.99; 95% CI, −7.88 to −0.83; *P* = .01). Unstandardized regression weights (*B*) are shown for every pathway in the diagram. Solid arrows denote significant associations. The dashed arrow denotes no significant association.

Figure 3 shows a graphical depiction of mediation analysis performed on data collected for precuneus Aβ deposition, nocturnal awakenings, and MMSE performance. We chose to focus on Aβ deposition in the precuneus for mediation analysis because of the particular vulnerability of the precuneus to Aβ accumulation,^33^ its emerging role in modulating slow-wave sleep,^34,35,36^ and our empirical finding that Aβ deposition in the precuneus had the strongest association with nocturnal awakenings. We found via mediation analysis that Aβ and MMSE performance were not directly associated (*B* = −1.55; 95% CI, −6.42 to 3.32; *P* = .50), consistent with the correlational data presented in Figure 2. However, when nocturnal awakening was introduced to the analysis as part of an indirect pathway, a statistically significant association between Aβ and poor MMSE performance was detected (*B* = −5.54; 95% CI, −10.24 to −0.85; *P* = .02). Finally, by comparing the strength of association between Aβ and poor MMSE performance in the direct vs indirect pathways, we found that nocturnal awakenings mediated the association between Aβ and poor MMSE performance (*B* = −3.99; 95% CI, −7.88 to −0.83; *P* = .01).

## Discussion

In patients with cognitive disorders, daytime sleepiness was associated with Aβ burden in the brainstem, whereas nocturnal awakenings were associated with Aβ burden in the precuneus. Importantly, only nocturnal awakenings were associated with poor performance on the MMSE. Moreover, an indirect association between Aβ and cognitive impairment was found to rely on nocturnal awakenings as an intermediary.

The associations of different sleep behaviors with Aβ deposition in specific brain regions are worth noting. For example, the association between daytime sleepiness and Aβ deposition in the brainstem of individuals with cognitive impairment has not been previously reported to our knowledge. Given the important role of the brainstem in attention and arousal,^37,38^ future investigation of the downstream consequences of brainstem Aβ deposition is warranted. Moreover, nocturnal awakenings exhibited a significant association with Aβ deposition in the precuneus, an interesting finding to consider in light of recent evidence demonstrating the role of the precuneus in facilitating slow-wave sleep^34,36^ and the implications of default-mode network dysfunction in sleep disruption.^35^ Future elucidation of how precuneus abnormalities intersect with sleep and cognition is likely vital to improving understanding of disease processes that underlie various dementia syndromes.

Previous studies have demonstrated that individuals with normal cognition and excessive daytime sleepiness have increased risk of cortical Aβ accumulation and cognitive decline.^10,19^ These associations were not observed in the present study, which may, in part, reflect the limitations of the MMSE in detecting subtle cognitive changes that occur in early stages of dementia. Therefore, future evaluation with more comprehensive neuropsychological testing is needed. Another possibility to consider is that a mechanistic shift may occur during the transition from normal aging to disease. In other words, factors leading to daytime sleepiness, which are likely important for the development of dementia in individuals with normal cognition, may evolve after the onset of dementia to have more complex associations with cognitive decline.

The finding that nocturnal awakenings are negatively associated with MMSE performance is consistent with previous polysomnographic data showing that wakefulness after sleep onset is associated with cognitive impairment.^39^ In addition, the results of this study provide novel insight by highlighting the role that Aβ may play in nocturnal awakenings and demonstrating that the association of Aβ with cognitive impairment relies on nocturnal awakenings as an intermediary. The indirect association of Aβ with cognitive impairment in patients with cognitive disorders may be important to consider given the failures of antiamyloid immunotherapy to improve cognition in clinical trials.^40^ Our data suggest that a combinational approach targeting both Aβ and sleep dysfunction may be necessary. Notably, recent studies^41,42^ indicate that tau may be a proximal mediator of sleep dysfunction, suggesting that therapeutic strategies targeting both Aβ and tau may be beneficial. Data from animal models of disease^18,43^ also demonstrate the contribution of corticothalamic circuit dysfunction to wakefulness after sleep onset, a mechanism that needs to be further explored in humans.

### Limitations

Although mediation analysis is a powerful tool that can disentangle direct and indirect associations among multiple variables of interest, an important point to keep in mind is that the analysis uses observational data to draw pathways of association, not causation. In particular, mediation analysis does not assess for the presence or absence of unmeasured variables, which can potentially be confounding and limit interpretation of causality. Therefore, the results of this study should only be interpreted as observational and associative, and a future interventional study would be needed to establish a causative pathway between Aβ, nocturnal awakenings, and cognitive impairment. We also acknowledge that the amount of time elapsed between MMSE testing and sleep questionnaire administration is a potential source of variability in the study. Other limitations include not assessing a wider range of sleep behaviors, such as napping. Sleep disorders other than sleep apnea were also not assessed. The lack of objective sleep data in this study precluded detailed evaluation of the effect of nocturnal awakenings on different sleep stages. Future studies should evaluate the relative contributions of rapid eye movement and non–rapid eye movement sleep disturbances to cognitive impairment.^16,39,41^ Longitudinal studies with larger sample sizes are also necessary to explore how sleep dysfunction evolves over time.

## Conclusions

In elderly individuals with cognitive disorders, Aβ accumulation is indirectly associated with cognitive impairment via nocturnal awakenings as an intermediary. Future investigation of the mechanism underlying this indirect association may be crucial for improved understanding of cognitive decline in disorders associated with Aβ accumulation.

## Notes

Supplement. **eAppendix.** Sample Sleep Questionnaires Form **eFigure.** β-Amyloid (Aβ) Deposition Across Regions of Interest (ROIs) in the Brains of Study Participants **eTable.** Regression Analysis of Nocturnal Awakenings vs β-Amyloid Deposition in Various Regions of Interest (ROIs)
