# Supplementary material for: Association of β-Amyloid Burden With Sleep Dysfunction and Cognitive Impairment in Elderly Individuals With Cognitive Disorders
Source: JAMA Netw Open. 2019 Oct 16;2(10):e1913383. doi: 10.1001/jamanetworkopen.2019.13383 (PMC6806437; doi:10.1001/jamanetworkopen.2019.13383)
Supplement: Supplement. — eAppendix. Sample Sleep Questionnaires Form eFigure. β-Amyloid (Aβ) Deposition Across Regions of Interest (ROIs) in the Brains of Study Participants eTable. Regression Analysis of Nocturnal Awakenings vs β-Amyloid Deposition in Various Regions of Interest (ROIs) [file jamanetwopen-2-e1913383-s001.pdf]

Supplementary Online Content

You JC, Jones E, Cross DE, et al. Association of β-amyloid burden with sleep dysfunction and cognitive impairment in elderly individuals with cognitive disorders. *JAMA Netw Open*. 2019;2(10):e1913383. doi:10.1001/jamanetworkopen.2019.13383

eAppendix. Sample Sleep Questionnaires Form

eFigure. β-Amyloid (Aβ) Deposition Across Regions of Interest (ROIs) in the Brains of Study Participants

eTable. Regression Analysis of Nocturnal Awakenings vs β-Amyloid Deposition in Various Regions of Interest (ROIs)

This supplementary material has been provided by the authors to give readers additional information about their work.

© 2019 You JC et al. *JAMA Network Open.*

eAppendix. Sample Sleep Questionnaires Form

Name:
Sex:
Age:
Date:

Nighttime Sleep Quality Survey

1. How long does it usually take for the you to fall asleep? _____ min
2. How long do you sleep every night on average? _____ hrs
3. How many times on average do you wake up in the middle of the night? _____ times
4. Do you have difficulty returning to sleep after waking up in the middle of the night? _____
    - Usually (3 or more times per week)
    - Sometimes (once or twice per week)
    - Seldom (less than once a week)
    - Never
5. Do you snore at night (yes or no)? _____
6. Have you ever been diagnosed with sleep apnea (yes or no)? _____
7. Do you use medication(s) to help with sleep (yes or no)? _____
    - If yes, which medication(s) and at what dose(s)? ___________________________
8. Rate your sleep quality from 0-10 (0 being terrible, 10 being excellent). _____

Modified Epworth Daytime Sleepiness Scale

In recent times, how likely are you to doze off or fall asleep in the following situations, in contrast to feeling just tired? If you are unlikely to be in one of these situations, try to work out how you would act based on your best understanding of your condition.

Use the following scale:
0 – would never fall asleep
1 – small chance of falling asleep
2 – medium chance of falling asleep
3 – high chance of falling asleep

Situations:

Sitting and reading ________
Watching TV ________
Sitting inactive in a public place (examples: theater or park) ________
As a passenger in a car for an hour without break ________
Lying down to rest in the afternoon, but not intending to sleep ________
In a conversation with someone (talking to or listening) ________
Sitting quietly after lunch without alcohol ________
Sitting down at a restaurant, waiting for the menu ________

© 2019 You JC et al. *JAMA Network Open.*

eFigure. β-Amyloid (Aβ) Deposition Across Regions of Interest (ROIs) in the Brains of Study Participants

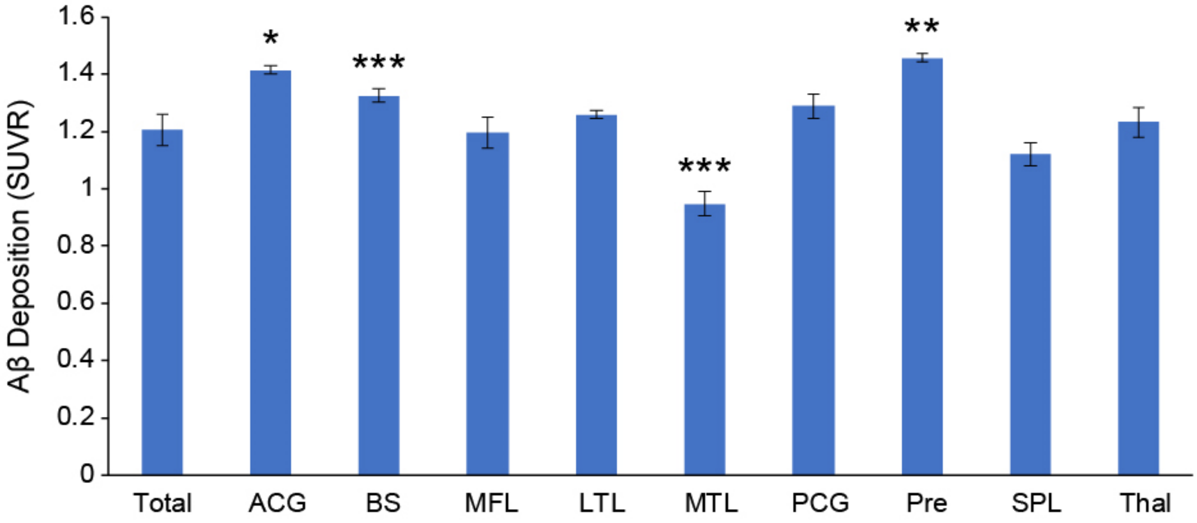

Total brain Aβ deposition (Total) represents the weighted average of Aβ deposition across all ROIs based on region size. ACG, anterior cingulate gyrus. BS, brainstem. MFL, medial frontal lobe. LTL, lateral temporal lobe. MTL, medial temporal lobe. PCG, posterior cingulate gyrus. Pre, precuneus. SPL, superior parietal lobe. Thal, thalamus. SUVR, standardized uptake ratio value. All comparisons shown are against total brain Aβ deposition. Error bars represent standard errors.

*$P < 0.05$.

**$P < 0.01$.

***$P < 0.001$.

© 2019 You JC et al. *JAMA Network Open.*

eTable. Regression Analysis of Nocturnal Awakenings vs β-Amyloid Deposition in Various Regions of Interest (ROIs)

ROI	*B*	95% CI	R^2	*P*
Anterior cingulate gyrus	0.095	0.031 to 0.160	0.152	0.004[a]
Brainstem	0.011	-0.007 to 0.028	0.029	0.22
Medial frontal lobe	0.074	0.026 to 0.122	0.162	0.003[a]
Lateral temporal lobe	0.073	0.024 to 0.121	0.153	0.004[a]
Medial temporal lobe	0.011	-0.006 to 0.028	0.013	0.21
Posterior cingulate gyrus	0.083	0.037 to 0.129	0.206	0.001[a]
Superior parietal lobe	0.059	0.02 to 0.098	0.141	0.004[a]
Thalamus	0.021	-0.002 to 0.044	0.046	0.254

Abbreviations: *B*, unstandardized regression weight; CI, confidence interval; R^2, coefficient of determination.
[a]Significant at the Bonferroni α = 0.006 (correcting for multiple comparisons).

© 2019 You JC et al. *JAMA Network Open.*
